# Iron Deposition in Parkinson’s Disease: A Mini-Review

**DOI:** 10.1007/s10571-024-01459-4

**Published:** 2024-02-23

**Authors:** Weiqi Zeng, Jin Cai, Lei Zhang, Qiwei Peng

**Affiliations:** 1 Department of Neurology, The First People’s Hospital of Foshan, Foshan, China; 2 Department of Cardiology, The Second Hospital of Zhangzhou, Zhangzhou, China; 3 Department of Neurology, Union Hospital, Tongji Medical College, Huazhong University of Science and Technology, Wuhan, China; 4 Department of Critical Care Medicine, Union Hospital, Tongji Medical College, Huazhong University of Science and Technology, Wuhan, China

**Keywords:** Neurodegeneration, Parkinson’s disease, Magnetic resonance imaging, Iron deposition, Iron chelation

## Abstract

Iron deposition is crucial pathological changes observed in patients with Parkinson's disease (PD). Recently, scientists have actively explored therapeutic approaches targeting iron deposition in PD. However, several clinical studies have failed to yield consistent results. In this review, we provide an overview of iron deposition in PD, from both basic research and clinical perspectives. PD patients exhibit abnormalities in various iron metabolism-related proteins, leading to disruptions in iron distribution, transport, storage, and circulation, ultimately resulting in iron deposition. Excess iron can induce oxidative stress and iron-related cell death, and exacerbate mitochondrial dysfunction, contributing to the progression of PD pathology. Magnetic resonance imaging studies have indicated that the characteristics of iron deposition in the brains of PD patients vary. Iron deposition correlates with the clinical symptoms of PD, and patients with different disease courses and clinical presentations display distinct patterns of iron deposition. These iron deposition patterns may contribute to PD diagnosis. Iron deposition is a promising target for PD treatment. However, further research is required to elucidate the underlying mechanisms and their impacts on PD.

**Graphical Abstract:**

Iron deposition in the Substantia nigra (SN) is a crucial pathological alteration in Parkinson's disease (PD). This article provides a review of the mechanisms and effects of iron deposition, as well as research on brain iron deposition in PD patients using magnetic resonance imaging (MRI). These findings elucidate the role of iron deposition in PD.

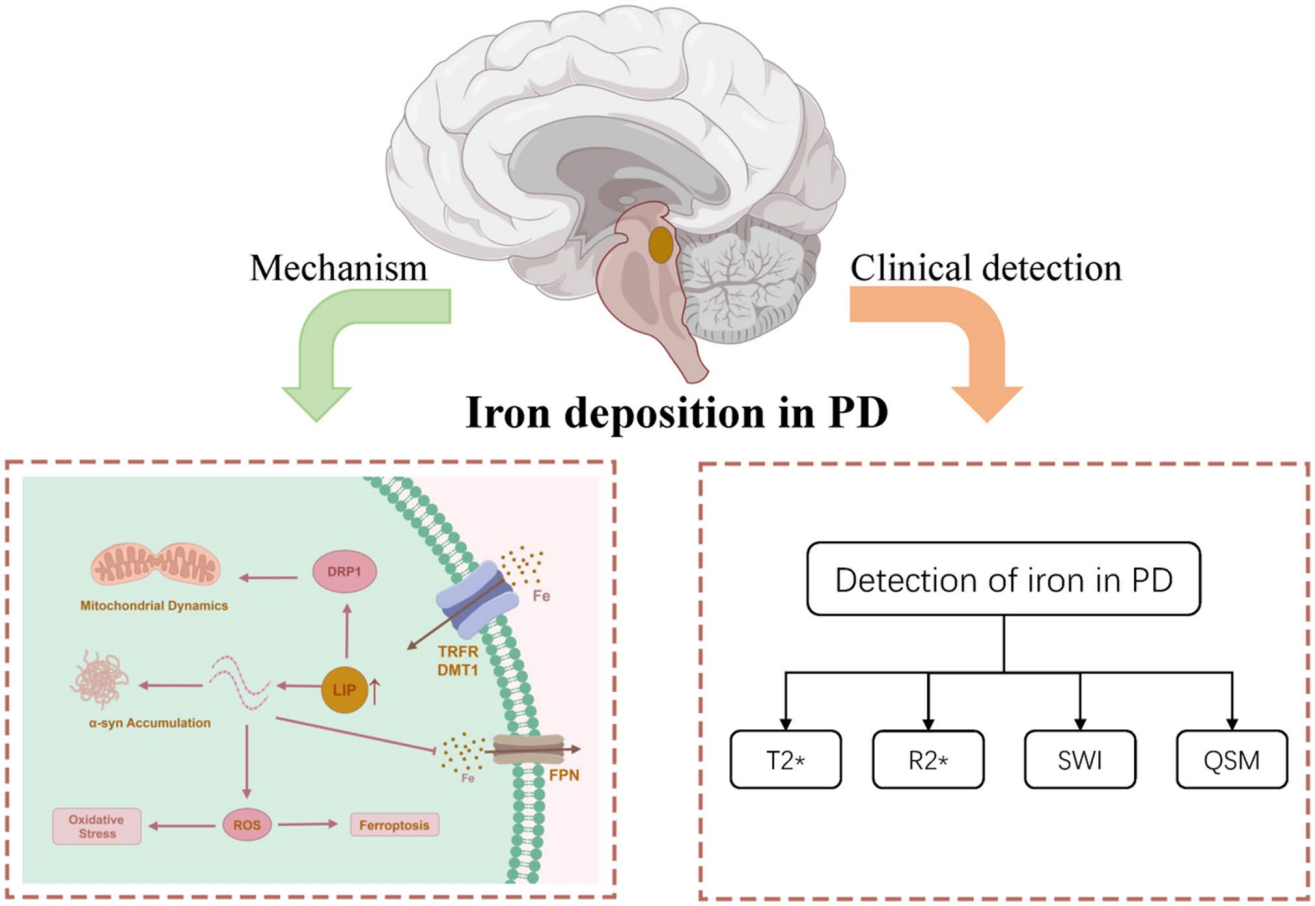

## Introduction

Parkinson's disease (PD) is a prevalent neurodegenerative disorder characterized by motor dysfunction including rigidity, tremors, and bradykinesia (Leite Silva et al. 2023). The precise etiological mechanisms underlying PD remain an area of ongoing research. Abnormal iron deposition in the substantia nigra (SN) is an important pathological change in PD; however, the mechanisms underlying iron deposition remain unclear. Iron plays a pivotal role as an indispensable cofactor in neuronal metabolic processes (Ward et al. 2014). Excessive iron or iron overload can trigger Fenton or Haber Weiss reactions, leading to the generation of reactive oxygen species (ROS), increased oxidative stress levels in tissues, cell damage, and ultimately cell death (Fischbacher et al. 2017). Abnormal iron deposition can also lead to the death of dopaminergic neurons in the SN through various pathways, such as by inducing mitochondrial dysfunction and promoting iron-dependent cell death (Zucca et al. 2017; Wang et al. 2022c).

Iron chelation therapy is a promising approach for the treatment of PD. Chelation therapy with iron chelators reduces brain iron deposition in PD patients and may improve motor dysfunction (Devos et al. 2014). However, in a recent international, multicenter, second-phase clinical trial involving patients with early PD, the FAIRPARK-II study (Devos et al. 2022) found that iron chelators worsened PD symptoms, suggesting that there are still many unanswered questions regarding iron deposition in PD.

In this mini-review, we will summarize the current findings on iron deposition in PD from both basic and clinical research perspectives and discuss potential mechanisms of iron deposition in PD, providing new insights into the treatment of iron metabolism disorders in PD.

## Iron Deposition and PD

### Mechanisms of Iron Deposition

Iron is an essential cofactor for maintaining the physiological functions of the nervous system. The central nervous system has developed a complex array of mechanisms to regulate and preserve iron homeostasis. Proteins including transferrin (TRF), divalent metal ion transporter 1 (DMT1), iron-regulatory proteins (IRP), and ceruloplasmin, participate in maintaining iron homeostasis. Furthermore, in instances of pronounced systemic iron deficiency, there is only a slight reduction in brain iron levels, indicating the potential role of the blood–brain barrier (BBB) in governing cerebral iron homeostasis (Youdim et al. 1989). Iron deposition may occur owing to disruptions in iron distribution, transport, storage, and circulation. Aberrant iron distribution forms the basis of iron deposition and transport, and storage abnormalities contribute to iron deposition in the SN.

It has become increasingly clear that iron deposition is intricately linked to various central nervous system disorders. Friedreich's ataxia, an autosomal recessive genetic condition characterized by mutations that result in frataxin deficiency. This deficiency disrupts iron homeostasis, leading to the accumulation of iron within the mitochondria, ultimately culminating in mitochondrial dysfunction (Tamarit et al. 2021). Neurodegeneration with brain iron accumulation is a group of progressive neurodegenerative diseases caused by iron salt deposition in the brain. This deposition occurs primarily within the basal ganglia, most notably in the globus pallidus and SN. The hallmark of this condition is progressive exacerbation of extrapyramidal symptoms and neurological manifestations, marked by significant clinical and genetic heterogeneity. Mutations in the genes associated with iron metabolism are frequently implicated in the onset of this condition (Hinarejos et al. 2020). Moreover, disorders of iron metabolism and deposition have been documented in hepatolenticular degeneration, Alzheimer's disease, hereditary spastic paraplegia, and PD (Gromadzka et al. 2021; Peng et al. 2021; Cosottini et al. 2022). The mechanisms underlying disruptions of iron metabolism exhibit distinct variations in various diseases. The subsequent sections provide a detailed exploration of the iron deposition mechanisms within the PD.

Abnormal iron distribution is the fundamental basis of iron deposition. Studies have demonstrated that individuals with PD, especially those with the HP 2–1 phenotype of haptoglobin (which is considered a risk factor for PD), tend to have decreased serum iron levels compared with healthy individuals (Costa-Mallen et al. 2015). The decrease in serum iron levels suggests that iron may be redistributed to other tissues such as the brain. Paola et al. examined the relationship between serum iron levels and iron content in specific brain regions, showing an association between increased iron content in the SN and decreased serum iron levels (Costa-Mallen et al. 2017). The reasons for this difference are still not fully understood; however, alterations in the BBB that result in disrupted iron transport may be a contributing factor. Alterations in the BBB of PD include an increase in endothelial cells in the SN pars compacta (Wada et al. 2006), increased integrin αvβ3 expression in the SN and globus pallidus (Desai Bradaric et al. 2012), microvascular degeneration, reduced and disrupted tight junctions, and changes in the microvascular basement membrane (Pienaar et al. 2015).

Abnormalities in iron transport may be one of the reasons for disrupted iron distribution. The central nervous system predominantly obtains iron via TRF and DMT1 (Jiang et al. 2017). Pathological α-synuclein proteins in PD can affect iron homeostasis by regulating the ubiquitination of DMT1 (Bi et al. 2020). Neuroinflammation induced by α-synuclein deposition results in a reduction in IRP and iron-exporting protein (FPN1) (responsible for iron efflux from cells) (Urrutia et al. 2013). Under neuroinflammatory conditions, both TRF receptors (TRFR) and DMT1 are upregulated, thereby facilitating iron uptake by brain cells (McCarthy et al. 2018). Lipocalin 2, an inflammatory response protein, mediates pathological iron transport in an inflammatory environment via the non-TRF pathway (Dekens et al. 2021). Ceruloplasmin, a ferroxidase, plays a crucial role in maintaining the activity of FPN1. In PD, reduced ceruloplasmin activity results in decreased FPN1 activity, restricting the efflux of cellular iron and contributing to iron overload (Jeong and David 2003). In contrast, a study reported that cerebral blood flow is reduced in patients with PD, which may also affect the efficiency of iron transport out of the brain, leading to elevated iron levels (Zhang et al. 2020).

Iron storage dysfunction in the SN is another significant factor contributing to iron deposition. Ferritin is a vital iron-binding protein, responsible for safely storing approximately 25% of the iron. The relationship between iron and metabolism in dopaminergic neurons led to the reasonable hypothesis that ferritin plays a role in regulating iron metabolism within these neurons. Additionally, ferritin serves as a pro-inflammatory molecule and acts as an inflammation marker, showing a strong correlation with the development and prognosis of various diseases (Zhang et al. 2021). In PD patients, a notable decrease in brain ferritin levels was observed, whereas total iron levels increased. This finding suggests the presence of unstable iron forms in the brains of PD patients (Dexter et al. 1991). Longitudinal studies have indicated a decline in ferritin levels in the cerebrospinal fluid as PD progresses, while total iron content increases (Zhang et al. 2021). In the SN of PD patients, the Fe^2+^/Fe^3+^ ratio increases, signifying the increased presence of unstable iron, which is often associated with more pronounced Fenton reactions (Wypijewska et al. 2010). Moreover, the ratio of heavy to light-ferritin chains is altered in PD. There is an increase in heavy chain ferritin coupled with a decrease in light chain ferritin, which leads to reduced stability of ferritin iron storage. This could be a contributing factor to the increase in unstable iron (Friedman and Galazka-Friedman 2012).

In the SN, iron predominantly resides in the neuromelanin (NM) of dopaminergic neurons. NM exerts neuroprotective effects through iron chelation, and approximately 50% of NM is normally present in an iron-chelated state. With advancing age, there is a gradual decline in NM levels in the SN (Zecca et al. 2001). In PD, the loss of dopaminergic neurons leads to a further reduction in NM content within the SN, resulting in increased levels of unstable iron (Aime et al. 2000). A PD cohort study has provided initial evidence confirming that the loss of dopaminergic neurons leads to disruptions in iron metabolism and iron deposition (Biondetti et al. 2021).

Additionally, the genetic variants rs602201 and rs198440 exert a stimulatory influence on nigral iron deposition in PD, suggesting that susceptibility to iron accumulation in the SN is partially genetically mediated (Wu et al. 2023).

### Iron Deposition and PD Pathology

Iron deposition significantly increases in the bilateral SN, red nucleus (RN), putamen, and globus pallidus of PD patients. Iron deposition is likely a common mechanism underlying microstructural alterations in the SN and putamen of PD patients (Yang et al. 2022). The dysfunction of dopaminergic striatal pathways and the loss of cells in the SN pars compacta are intricately linked to the elevated iron content in the SN (Biondetti et al. 2021). Iron deposition can activate microglia and astrocytes, promoting the release of pro-inflammatory cytokines and oxidative stress factors (McCarthy et al. 2018). This leads to neuroinflammation, which exacerbates the loss of dopaminergic neurons and accelerates the progression of PD.

The inflammatory state and the progression of chronic brain iron deposition in PD patients may occur independently. Changes in systemic inflammation were not found to be correlated with peripheral iron metabolism and may not play a role in the worsening of brain iron deposition in PD patients (Xu et al. 2022). In addition to the generation of ROS through the Fenton and Haber Weiss reactions, which increase oxidative stress levels, iron overload can damage the SN and accelerate the progression of PD through other mechanisms. If the concentration of ROS exceeds the capacity of antioxidants, it leads to substantial cellular harm, including DNA mutations, impairment of mitochondrial functionality, lipid peroxidation, and modifications to proteins (Jansen van Rensburg et al. 2021). These cumulative effects ultimately lead to oxidative stress and cell death.

Iron-dependent programmed cell death, also known as ferroptosis, has attracted considerable attention in recent years. Ferroptosis is commonly linked to the excessive intracellular accumulation of unbound iron ions and excessive ROS (Mahoney-Sánchez et al. 2021). Iron overload inevitably promotes ferroptosis, thereby accelerating the progression of PD. Given that numerous comprehensive reviews have extensively covered ferroptosis, this review will not delve into it further (Wang et al. 2022a, c; Dusek et al. 2022).

Significant iron deposition within the Lewy bodies has been identified in the remaining dopaminergic neurons of the SN in PD patients (Castellani et al. 2000). This finding suggests that iron deposition shares a common underlying pathological mechanism with the deposition of pathological α-synuclein. In vitro electron microscopy confirmed that Fe^3+^ could induce the formation of discrete alpha-synuclein fibrillar species (Bharathi et al. 2007). Fe^3+^ can directly bind to α-synuclein, altering its conformation (Peng et al. 2010). Additionally, iron can modulate the expression of α-synuclein at both the transcriptional and translational stages. The 5' untranslated region of human α-synuclein transcript contains a single stem-loop structure composed of 46 nucleotides, known as the iron-responsive element (IRE) (Friedlich et al. 2007). IRP, responsible for maintaining iron homeostasis, can promote the degradation of α-synuclein mRNA by binding to the IRE. In instances of elevated iron levels, IRP fails to engage with the IRE within the α-synuclein mRNA, resulting in an augmentation of α-synuclein expression and the subsequent onset of pathological aggregation (Li et al. 2011). Deferoxamine can reduce α-synuclein mRNA levels, providing further support for the role of iron in the post-transcriptional regulation of α-synuclein (Febbraro et al. 2012). α-synuclein undergoes diverse post-translational modifications subsequent to its translation, encompassing acetylation, oxidation, ubiquitination, nitration, glycosylation, and phosphorylation (Barrett and Timothy Greenamyre 2015). Importantly, certain post-translational modification processes are susceptible to the influence of iron deposition, resulting in heightened toxicity and aberrant aggregation of α-synuclein.

The deposition of iron influences mitochondrial dynamics, turning mitochondria towards fission, resulting in the generation of a greater number of fragmented mitochondria. Iron deposition in subcortical brain regions, specifically in the putamen and globus pallidus, has been linked to mitochondrial dysfunction in PD patients (Prasuhn et al. 2022a). In an environment characterized by elevated iron levels, the significant generation of ROS plays a pivotal role in the activation of the essential protein dynamin-related protein 1 (DRP1). DRP1, in turn, governs mitochondrial fission and facilitates mitochondrial division (Lee et al. 2018, 2020). Reducing oxidative stress levels has the potential to ameliorate mitochondrial fragmentation and mitigate neuronal degeneration (Kim et al. 2019). Elevated iron concentrations enhance calcium phosphatase activity, leading to increased intracellular calcium levels. Consequently, this heightened calcium influx activates DRP1, facilitating mitochondrial fission and contributing to the degenerative processes (Lee et al. 2016).

**Fig. 1:**
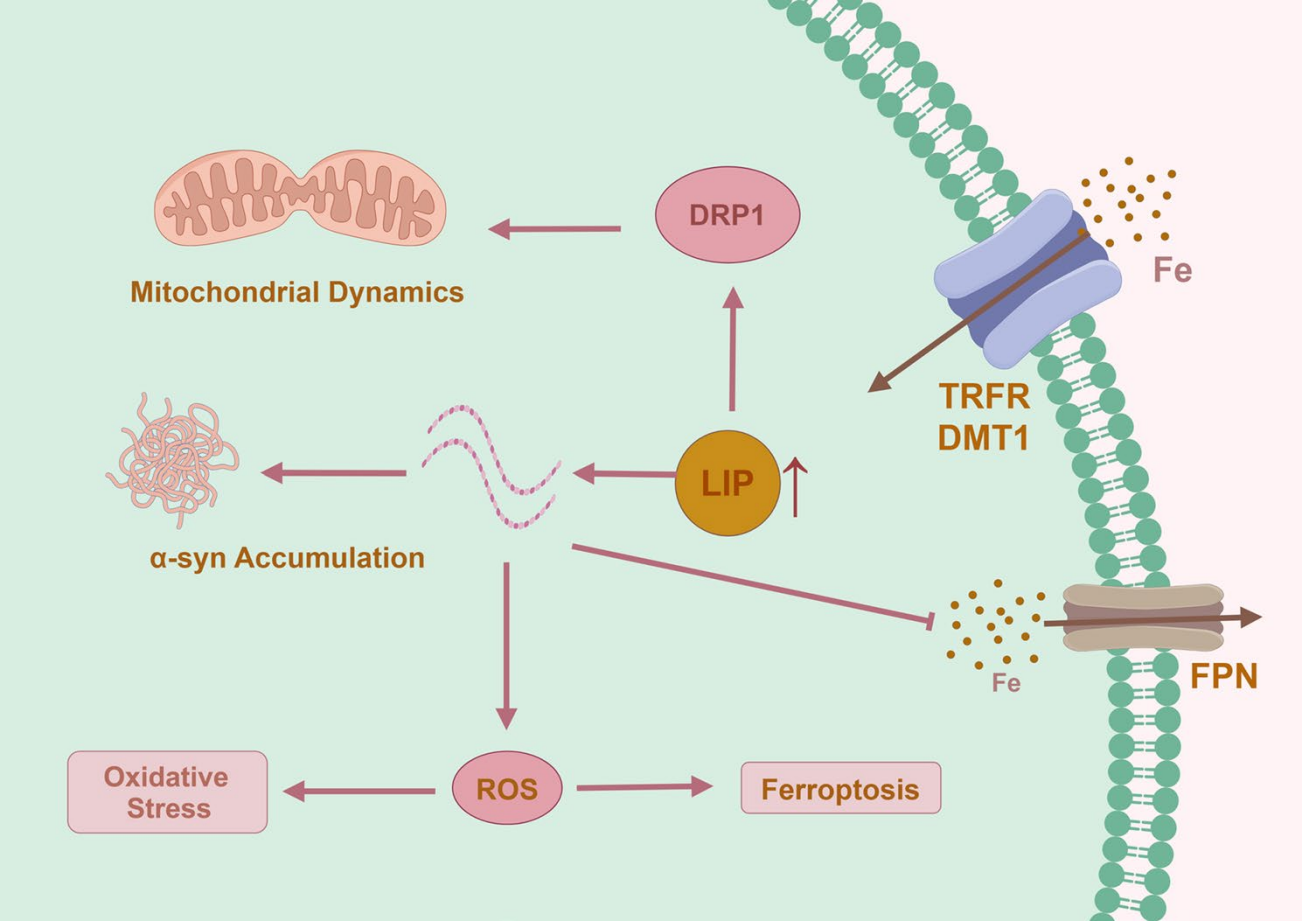
The impact of iron on brain cells in Parkinson’s disease. Iron (primarily in the form of transferrin) is transported into cells through transport proteins such as the transferrin receptor (TRFR) and divalent metal transporter 1 (DMT1). Excess iron leads to the elevation of the labile iron pool (LIP). The elevated LIP has the following effects: (1) activation of dynamin-related protein 1 (DRP1), promoting mitochondrial fission; (2) facilitation of α-synuclein deposition, increasing its cytotoxicity; and (3) increased oxidative stress levels, resulting in ferroptosis

Iron deposition promotes PD progression by inducing oxidative stress, iron-dependent cell death, exacerbating α-synuclein pathology, causing mitochondrial dysfunction, and triggering neuroinflammation. In conclusion, iron deposition in PD is a complex process involving multiple mechanisms, including alterations in iron distribution, transport, storage, and circulation, leading to iron deposition in the SN and other brain regions (Fig. 1).

Iron deposition occurs in many nuclei, but in some nuclei, despite the presence of iron deposition, the loss of neurons does not seem to be severe (Hare et al. 2015). This suggests that certain factors may be involved in iron deposition-mediated neuronal damage. Metabolites of dopamine are known to have neurotoxic effects, but the exact mechanism of how they damage neurons remains incompletely understood (Hurben and Tretyakova 2022). Some evidence suggests that, in conditions of elevated iron content, intermediate or final products of dopamine metabolism interact with iron, giving rise to neurotoxic substances. This phenomenon is referred to as iron-mediated dopamine neurotoxicity (Hare and Double 2016). Therefore, some refer to iron and dopamine as a toxic couple. The mechanisms involved in this process are complex, and for a more detailed exploration, readers are referred to Wise's review (Wise et al. 2022). This theory suggests a significant role for dopamine metabolism in the pathogenesis of PD, effectively explaining the neurotoxic mechanisms of dopamine metabolites.

## Iron Deposition in PD Patients

Increased iron deposition in the SN was observed during autopsy in patients with PD approximately half a century ago (Dexter et al. 1987). Nevertheless, owing to research constrains in this era, the association between SN iron accumulation and the pathogenesis of PD remains inconclusive. With the advent of technological progress, particularly the application of magnetic resonance imaging (MRI), it has become possible to non-invasively evaluate the levels of paramagnetic substances within the SN in vivo.

### Assessment of Cerebral Iron Deposition

Gradient echo T2*-weighted imaging can discern alterations in magnetic susceptibility, and was initially used to detect paramagnetic substances intracranially. This sequence has been used to identify iron deposition in the brains of PD patients (Gorell et al. 1995). However, conventional T2*-weighted imaging is two-dimensional, and its slice-direction resolution is compromised after three-dimensional reconstruction. However, the sensitivity to iron deposition is influenced by several factors. Additionally, a constraint of this methodology is its inability to perform quantitative analysis.

Susceptibility-weighted imaging (SWI) is a technology based on T2*-weighted imaging, which allows imaging based on the differential magnetic susceptibility of different tissue components. This provides a straightforward and efficient method for the detection of iron deposition. In SWI images of healthy individuals, nigrosome 1 (N1) in the SN exhibits a high signal intensity, flanked by low-signal areas on the front and sides, resembling the tail of a swallow, which is referred to as the “swallow tail sign”. In contrast, in PD patients, owing to a significant reduction in N1 content and increased iron deposition, the swallow tail sign becomes blurry or disappears (Mueller et al. 2014). SWI is a three-dimensional imaging technique with significantly improved spatial resolution compared with T2*-weighted imaging. However, this is a qualitative detection method that cannot provide quantitative information.

R2*-weighted imaging addresses the challenge of quantifying the iron content in the SN. It is based on traditional MRI for iron quantification and utilizes the transverse magnetization rate (R2*) at the pixel level to quantify iron. In the early stages of PD, the R2* value of the SN exhibits only a mild elevation. However, as the PD progresses, the R2* value increases gradually (Hopes et al. 2016; Du et al. 2018). Nevertheless, R2*-weighted imaging relies solely relies on the amplitude information in MRI signals and disregards the phase information from the differences in magnetization between tissues. Post-mortem examinations have confirmed that R2* can reflect the iron content in different brain regions, indicating that MRI technology can be utilized to assess the iron content in tissues (Brammerloh et al. 2021).

Quantitative Susceptibility Mapping (QSM) effectively overcomes the limitations of R2*-weighted imaging, allowing the calculation of magnetization rate values at the pixel level and providing more accurate information on tissue iron. After continuous improvements, QSM has significantly overcome challenges associated with “background field interference” and the “inverse problem of field-to-source mapping” (Pyatigorskaya and Santin 2021). QSM demonstrates heightened sensitivity in quantifying tissue iron levels, accompanied by enhanced reliability and repeatability (Pyatigorskaya and Santin 2021). PD patients exhibited significantly increased QSM values in the SN, RN, globus pallidus, and putamen. Iron content in the SN and paleostriatum gradually increased throughout the disease course and was associated with clinical features (Fu et al. 2021). QSM reveals the temporal changes in iron content of N1. Quantification of iron holds the potential to serve as a valuable biomarker for assessing neurodegeneration during the prodromal stage of PD (Lancione et al. 2022). R2* and QSM also demonstrate correlations with both glial density and tau burden (Wang et al. 2023).

Multi-parametric MRI sequences are valuable for assessing iron deposition in the substantia nigra pars compacta in PD. It reveals the correlation between iron deposition and the decline in dopamine function and reduced neuromelanin in the SN of PD (Depierreux et al. 2021). The quantitative evaluation of iron accumulation within the inferior region of the SN pars compacta holds the potential to function as an imaging biomarker for the early diagnosis, severity assessment, and subsequent monitoring of PD (Cao et al. 2022).

### MRI Findings of Iron Deposition in PD Patients

Exploring specific brain regions where iron deposition occurs in PD patients is a topic of significant investigative interest. In comparison to other brain regions, the SN is the sole structure that displays higher susceptibility to abnormal iron deposition (Ghassaban et al. 2019). Compared to the younger, the older exhibit significantly higher iron deposition. In the prodromal stages of PD, spatial variations in iron distribution within the subcortical nuclei, aside from the SN, are more pronounced. From the prodromal stage to clinical PD, iron deposition gradually increases in the SN and RN (Zang et al. 2022). A three-year follow-up study involving 18 patients with PD reported that iron deposition in PD predominantly occurs within the ventral region of the SN (Bergsland et al. 2019). In addition to the SN, the globus pallidus and the subthalamic nucleus are also prominent sites of iron deposition (Martin-Bastida et al. 2017a). In contrast, research has also suggested that in comparison to the control group, the increase in iron within the SN of PD patients is not significantly pronounced(Dashtipour et al. 2015). This could be attributed to the insensitivity of the detection methods and relatively small sample size.

The relationship between iron deposition and the progression of PD remains unclear. Zhang et al. revealed that during the prodromal and early clinical stages of PD, there is no observable augmentation in iron accumulation within the ventral tegmental area. However, iron deposition exhibits a substantial increase as PD progresses into more severe stages (Zhang et al. 2023a). A two-year follow-up study indicated a negative correlation between iron deposition and dopamine transporters in early PD patients (Biondetti et al. 2021). However, another study suggested that iron deposition in the SN was not associated with disease severity (Cheng et al. 2020). A study involving a 36-month follow-up of early PD patients found no significant changes in iron deposition in the SN (Wieler et al. 2015). There were neither significant difference in parameters based on neuromelanin-weighted imaging and SWI between PD patients and healthy controls. There was no significant association observed between the extent of midbrain hyperechogenicity and indices derived from either neuromelanin-weighted imaging or SWI (Prasuhn et al. 2022b).

Iron deposition may also be related to levodopa use. Initiating timely and consistent administration of antiparkinsonian medications mitigates the accumulation of iron within the N1 (Wang et al. 2022b). Administration of levodopa therapy exacerbates iron deposition in PD patients, suggesting that elevated iron deposition may result from the medication rather than the disease itself (Du et al. 2022). Notably, this study also observed a relationship between iron deposition and selegiline use, with milder SN iron deposition associated with selegiline administration. This finding suggests a potential disease-modifying effect of selegiline (Du et al. 2022).

As PD progresses, iron deposition is more pronounced in certain brain regions. Among patients with advanced PD, there is a more serious iron deposition in the SN pars compacta and globus pallidus (Guan et al. 2017). The extent of iron deposition was negatively correlated with the Hoehn–Yahr stage (Wu et al. 2014). Some researchers believe that iron deposition may not increase gradually with disease progression but could potentially increase abruptly at a certain stage (Dusek et al. 2022). However, this hypothesis requires further confirmation through larger clinical studies.

### Iron Deposition and PD Symptoms

The extent of iron deposition is associated with the progression of PD. Various levels of iron deposition have been noted within the SN pars compacta and reticulata in both early- and late-onset PD. Iron accumulation takes place at an early stage of PD and is limited to the SN and RN (Li et al. 2022). However, iron deposition in the putamen is exclusive to patients with late-onset PD. Notably, there was no correlation between the extent of iron deposition and the clinical symptoms in early-onset PD (Xuan et al. 2017).

Iron deposition exhibits regional selectivity in the cortical brain regions of PD (Thomas et al. 2021). Distinct iron deposition patterns were observed within the basal ganglia when comparing the postural instability and gait difficulty and tremor dominant groups. Additionally, variations in iron accumulation appear to exist among PD motor subtypes with respect to the major motor symptom sides (Zhang et al. 2023b). Different clinical subtypes of PD exhibit distinct patterns of SN iron deposition, suggesting that iron deposition plays a role in the pathogenesis of various PD subtypes (Xiong et al. 2020). A study employing transcranial sonography found that PD patients with SN and hyperechogenicity experienced more severe motor disturbances, accompanied by elevated serum TRF and cerebrospinal fluid iron levels (Yu et al. 2018). This finding implies an association between SN iron deposition and clinical manifestations of PD.

A study using QSM revealed that PD patients with more serious SN iron deposition often presented with higher Unified Parkinson's Disease Rating Scale (UPDRS) motor scores (Ghassaban et al. 2019). After adjusting for age and gender, there is a significant correlation between MDS-UPDRS III scores and putamen iron deposition. The combination of QSM and diffusion kurtosis imaging demonstrates good diagnostic efficacy in early PD (Tan et al. 2021). Another study combining magnetic resonance spectroscopy (MRS) and R2* sequences confirmed the association between motor dysfunction in PD and alterations in brain iron content and neuro-metabolites (Pesch et al. 2019). The dysfunction of the basal ganglia network and visual hyperfunction in PD is related to iron deposition (Wen et al. 2022). In addition, iron deposition in the dentate nucleus of the cerebellum may be associated with to PD tremors (He et al. 2017). In PD patients with levodopa-induced dyskinesia (LID), iron deposition in the SN is higher compared to those without LID. The QSM values of the SN could potentially serve as early diagnostic neuroimaging biomarkers for LID (Song et al. 2021).

Rapid eye movement sleep behavior disorder (RBD) is considered a risk factor for PD. Brain iron deposition measurements in RBD patients have shown increased iron content in the bilateral SN, although this was lower than that observed in PD patients. However, further research is needed to determine whether increased iron content increases the risk of RBD in patients with PD (Sun et al. 2020). The iron content in the extra-basal ganglia system is associated with non-motor symptoms of PD, particularly sleep issues and autonomic dysfunction (Kim et al. 2021).

Iron deposition is also associated with Parkinson's disease dementia (PDD). PDD patients exhibit significantly higher magnetization transfer values in both hippocampi than healthy controls and non-demented PD patients (Li et al. 2018). Moreover, this elevated magnetization transfer was inversely correlated with cognition. In certain regions of the white matter, primarily in the corpus callosum and left hemisphere, a significant positive correlation was observed between R2* values and Montreal of Cognitive Assessment scores (Kan et al. 2022). Iron deposition in the hippocampus and thalamus of PD patients is linked to lower cognition, whereas iron deposition in the putamen is associated with higher motor impairment scores (Thomas et al. 2020). Assessment of brain tissue iron content via QSM can be employed to monitor and track alterations in clinical symptom in PD patients (Thomas et al. 2020). Few studies have explored the correlation between iron deposition and non-motor symptoms. One small-scale study found that increased R2* values in the RN and right amygdala are related to PD sleep disturbances, whereas elevated R2* values in the right amygdala and left hippocampus are associated with PD autonomic dysfunction (Kim et al. 2021). Additionally, anxiety in PD is linked to increased iron accumulation within the neural circuits associated with fear in the brain (Chen et al. 2023).

### Screening and Differentiating PD through Brain Iron Deposition

A significant increase in the iron content within the SN of PD patients contributes to PD screening. Iron accumulation in the brains of healthy individuals typically exhibits an age-related increase. However, this increase is substantially lower than that observed in PD patients (Buijs et al. 2017; Lu et al. 2017). The magnetic susceptibility and kurtosis in the SN are potential indicators of early stage PD (Rong et al. 2022). Discernible SN iron deposition in PD offers the potential for PD screening in larger populations (Ghassaban et al. 2019).

Patients with atypical PD symptoms must be distinguished from atypical parkinsonism, such as multiple system atrophy (MSA) and progressive supranuclear palsy (PSP). Atypical parkinsonism also often exhibits brain iron deposition, but distinct patterns of iron accumulation can aid in distinguishing PD from other degenerative diseases. In comparison to patients with PD, patients with MSA exhibit more serious iron deposition in the putamen and pulvinar thalamus, with the lower inner region of the putamen displaying the most distinct iron deposition (Wang et al. 2012). Increased iron deposition within the RN is a supportive diagnostic feature of PSP (Zhang et al. 2022). Furthermore, the iron within the PSP brain may consist of hemosiderin, rather than ferritin (Foroutan et al. 2013). Combining plasma neurofilament light chain (NfL) levels with iron deposition measurements can significantly enhance the precision of differentiating among PD, MSA, and PSP (Zhang et al. 2022).

### Iron Chelation Therapy in PD

Exploratory treatments targeting iron deposition in PD patients have also been investigated. Deferiprone (DFP) is an iron chelator capable of crossing the BBB to chelate unstable iron pools within brain cells (Cabantchik et al. 2013; Devos et al. 2014). A pilot, prospective, randomized, double-blind trial including 37 PD patients, evaluated the therapeutic effects of DFP on PD using R2* and motor impairment scores. These results demonstrate that iron chelation therapy could alleviate SN iron deposition and slow the progression of motor impairments (Devos et al. 2014). Another study involving 22 early-onset PD patients found that a 6-month DFP treatment reduced iron content in the dentate and caudate nuclei (evaluated by T2*), showing trends of improvement in motor impairment and quality of life scores, although no significant differences were observed compared to the control group (Martin-Bastida et al. 2017b).

An international multicenter Phase II clinical trial on de novo PD, known as the FAIRPARK-II study, included 372 patients who were allocated in a 1:1 ratio to the experimental and control groups. After 36 weeks of intervention, the patients in the experimental group had worse motor impairment scores than those in the control group, leading to a negative result (Devos et al. 2022). The study did not achieve the positive results, indicating that further research is needed to investigate the role of iron deposition in the pathological mechanisms of Parkinson's disease.

PD is a highly heterogeneous condition, and the contribution of iron deposition to the pathogenesis of PD may vary among different individuals. Therefore, the timing and selection of individuals for iron chelation therapy requires further exploration. The preceding text has elaborated on the association between iron deposition and neuronal damage, which is relevant to the clinical symptoms and disease progression. However, excessive chelation of iron can affect the function of the dopamine system (Unger et al. 2008). Engaging in iron chelation therapy without any assessments may impact tyrosine hydroxylase and dopamine synthesis; the expression and function of dopamine D2 receptors are also closely linked to iron. Currently, the understanding of iron deposition in PD largely origins from imaging-based studies, while the specific mechanisms underlying iron deposition remain unclear. The broad-spectrum iron chelation therapy applied in the FAIRPARK-II study may have some negative effects. Clarifying the pathological mechanisms of iron deposition and exploring targeted iron chelation or inhibition of iron deposition could represent a novel therapeutic approach.

## Future Prospects

Recently, iron deposition in PD has garnered increasing attention and research efforts. It exacerbates the pathological progression of PD through various mechanisms and is a critical component of the vicious cycle of PD pathology (Song and Xie 2018). The failure of the FAIRPARK-II study (Devos et al. 2022) underscores our incomplete understanding of the mechanisms underlying iron deposition in PD. The FAIRPARK-II study seems to suggest that iron is a double-edged sword, with both excessive and insufficient levels being harmful. The correction of iron imbalance still requires further exploration. Therefore, further research is warranted to elucidate the role of iron deposition in PD and provide valuable insights into the development of PD therapies targeting iron deposition.

## Data Availability

Data availability is not applicable to this article as no new data were created or analyzed in this study.
